# Perceptions About Disseminating Health Information Among Mommy Bloggers: Quantitative Study

**DOI:** 10.2196/resprot.7764

**Published:** 2018-04-24

**Authors:** Amelia Burke-Garcia, Gary L Kreps, Kevin B Wright

**Affiliations:** ^1^ Westat Rockville, MD United States; ^2^ Department of Communication George Mason University Fairfax, VA United States

**Keywords:** mommy bloggers, social media, health messages, health information dissemination

## Abstract

**Background:**

Social media are potentially powerful channels for communicating relevant health information in culturally sensitive and influential ways to key audiences. Moreover, these channels hold promise for promoting awareness and knowledge of health risks, prevention, and treatment by utilizing opinion leaders for message dissemination. Despite limited empirical evidence to-date, early promising results suggest that blogs are a form of social media that should be examined as worthy channels for health communication.

**Objectives:**

This formative study explored mommy bloggers’ perceptions about sharing health-related information on their blogs with their readers. It also sought to analyze which topics would be of most interest to mommy bloggers, what motivates them to write about health issues, and how they perceive interest in these topics among their readers.

**Methods:**

This study employed survey methodology, including the use of open-ended questions, the responses to which were coded for analysis. Specifically, a 14-item survey was fielded with mommy bloggers between October 1 and October 28, 2016. Bloggers were recruited through The Motherhood network. A total of 461 mommy bloggers responded to the survey; 163 were removed for low quality responses and incomplete data. As a result, 298 eligible participants completed the survey. For open-ended questions in the survey, a sample of responses were coded and analyzed.

**Results:**

The majority of the respondents (87.2%, 260/298) reported that they have written about health issues in the past; 97.3% (290/298) of the respondents reported that they would consider writing about health issues sometime in the future, and 96.3% (287/298) of the respondents reported that their readers like to read about health issues on their blogs. In terms of content priorities for this sample of bloggers, Nutrition and Physical Activity dominate the current conversation and similarly, Physical Activity and Nutrition remain top content priorities for these bloggers for the future. Moreover, 21.3% of the respondents reported that their readers would be interested in these topics. Finally, having a personal connection with a health issue was found to be positively associated with likeliness to write about health issues on their blog (*P*<.001).

**Conclusions:**

This study illustrates that there are potentially rich opportunities for working with mommy bloggers to communicate with key health decision makers (moms) on important health issues. There is a great support among mommy bloggers for health information dissemination as well as interest for accessing relevant health information from their readers. This presents an opportunity for public health research and communication campaigns to more broadly promote their messages, thereby contributing to their behavior change objectives. Limitations included overrepresentation of white, higher-educated, and younger women. It suggests a need for more targeted engagement of a diverse sample for future work.

## Introduction

Health is nothing if not complex [1], and health communication is the “crucial social process for enabling health care consumers and providers to manage the complex information demands of health care and health promotion, especially in times of high uncertainty” [2]. Health communication has been a primary strategy to improve people’s health [3]. Historically, health communication has focused on disseminating evidence-based messages from experts to the public in the hope of motivating them to adopt healthy behaviors and use health care effectively [3,4]; however, these top-down communication approaches have not been particularly effective in engaging key audiences and increasing their understanding and adoption of key health recommendations [5-7]. Problems associated with these traditional communication methods include limited message exposure, failure to target and tailor messages to consumers’ literacy levels and cultural orientations, and limited audience access to and use of the communication channels employed [8,9].

Recent studies have shown that Americans are increasingly using the Internet as a primary health information source, with 8 in 10 Internet users in the United States now using the Internet to find health information [10]. Moreover, increasing importance is being placed on the Internet than on interacting directly with providers as an information source [11]. Compounding this is the fact that social media use has vastly increased in the last 10 years, with 65% of American adults now using social networking sites [12]. Much of this use is focused on health-related information dissemination and engagement [12], as social media contribute to “facilitating, sharing, and obtaining health messages” [13].

For over a decade, consumers have been increasingly seeking active channels such as the Internet as a primary source of health information—particularly for the most health conscious [14]. In terms of health information, 86% of women report that they make the decisions about health care treatments for their entire family [15]; and Bailey posits that health communicators should go where women spend time, which increasingly means online social network and blogging sites [16].

The dialogic nature of social media allows senders to reach broad audiences to participate in Web-based conversations about health issues. A few studies have examined health promotion through social media [17-22], the findings from which indicate great potential for using blogs, Facebook, Twitter, and other Web-based communication channels not only for increasing awareness but also to influence decision making concerning health issues. Specifically, blogs represent one powerful channel for potential exploration of health communication messaging and strategies. Blogs are rich communication channels, with different types of information that are shared interactively via bloggers’ posts and exchanges of comments from blog readers [23]. The activity on these blogs—both by bloggers and blog readers—create and share information at an unprecedented level, resulting in the broad dissemination of text-based, photo, and video content [23]. Imbued “with a strong sense of the author’s personality, passions, and point of view” [24], blogs result in opinions and ideas being shared online [23]. This community therefore has a virtual finger on the pulse of the needs and beliefs of Web-based audiences [23]. In this way, blogs, and their authors, or bloggers, act as opinion leaders for their readers. Yet, as Sundar et al contend [25], “Relatively little media and scholarly attention has been paid to personal blogs that discuss topics other than politics. For example, the burgeoning number of health blogs has gone virtually unnoticed in mainstream media and the academic discourse about blogs.”

A subgroup of blogs called mommy blogs has become popularized in recent years. There are about 3.9 million moms in the United States who identify as bloggers [26]. A mommy blogger is defined as, “A mother who blogs about her children, motherhood, parenting or related topics” [27]. The average mommy blogger is 37 years old, and almost 90% of mommy bloggers have kids between the ages of 2 and 11 [26] (It is worth noting that there are also daddy bloggers, and these comprise approximately 3% of all bloggers [28]). Mommy bloggers have traditionally been white, middle to upper class, educated mothers with the average mommy blogger’s household income ranging between US $14,000 and US $84,000, which is higher than the average income level for nonblogging moms [26]. In addition, moms who read or contribute to blogs are also 52% more likely to have college degrees than moms who do not blog [26]**.** This suggests that, “mommy bloggers belong to a pretty elite social set” [26]; however, increasingly, mommy bloggers are becoming more diverse [29].

Ultimately, with 14% of all American mothers with at least 1 child in their household turning to blogs for advice [26], these kinds of blogs can be important sources of information about a variety of topics. Parenting websites are the top source moms use to learn about products and services [30], but these blogs can also serve as sources of social support, connection, and validation for women navigating important health decisions for themselves and their family [31-33]. As a one-stop shop for entertainment, creative ideas, and useful information (including health), it is no surprise that mommy blogs have become so popular. Some of the most successful mommy bloggers tout thousands of followers and readers and earn their living through blogging [34]. Seen as a trusted member of this virtual community, their readers rely upon them for news and information [35]. This presents an opportunity for public health research and communication campaigns to more broadly promote their messages, thereby contributing to their behavior change objectives. The value of these mommy blogs should not be overlooked by public health communicators and should be further explored as sources of interpersonal influence concerning important health issues.

Despite mommy bloggers wielding strong influence with their readers, little research has focused on these bloggers as channels for communication concerning health promotion initiatives. Research by Burke-Garcia et al included formative interviews with mommy bloggers, followed by an intervention to get these bloggers to post health information on their blogs [36]. Data from the study revealed that bloggers can drive health behavior consideration among their readers, but there are also barriers among bloggers concerning sharing of certain kinds of health information [36]. Additionally, Horn et al conducted a pilot study using mommy bloggers to reach communities of color with health information, which found that the collaboration between health professionals and social media leaders in communities of color has the ability to influence content and dialogue and thus can help reduce health disparities [37].

This paper reports a formative research study that aimed to explore the full complexity of mommy bloggers’ perceptions about writing about health-related topics on their blogs in order to help inform future public health initiatives that may seek to use these layperson Web-based opinion leaders. It builds on prior research that looks at the use of Web-based sources as being influential to health information dissemination [33] and expands the limited body of work on mommy bloggers as powerful communication channels for health that exists currently. Findings include an overall willingness to write about health topics among this audience and that mommy bloggers’ personal history of having health issues is positively linked with increased likelihood to write about health issues on their blog. This paper reviews the study design and methods as well as the findings and future research opportunities. Finally, it acknowledges the limitations of this study, which were a lack of opportunity to gain additional insight through interviews with the bloggers themselves and the use of a convenience sample, which resulted in the respondents skewing white, female, educated, and younger and which may have influenced the results.

## Methods

### Research Questions

This pilot study attempted to capture the complexity of mommy bloggers’ perceptions about writing about health-related topics on their blogs. This study utilized an inductive approach and purposive sampling whereby, a nonprobability sample was obtained based on the characteristics of the population studied and the objective of the study [38] to explore bloggers’ perceptions about writing about health-related topics on their blogs. Specifically, the researcher hoped to better understand mommy blogger perspectives about which issues are more likely to be written about and their motivations for doing so, as well as the perceived interest among their readers for consuming health-related content. In pursuit of these goals, the following research questions were posited:

RQ1. How willing are mommy bloggers to write about health issues on their blogs?

RQ2. How do bloggers perceive the interest of their readers to read about health issues on blogs?

RQ3. Which topics are mommy bloggers most interested in writing about?

RQ4. How do bloggers perceive the health topic preferences of their readers?

RQ5. What motivates mommy bloggers to write about health issues?

RQ6. What factors contribute to mommy bloggers writing about health issues?

While this is a highly formative study, the researchers hoped to glean some insights that may serve to expand research in this area and inform a larger study and possibly future health communication campaigns.

### Recruitment

Institutional Review Board (IRB) approval was obtained through George Mason University’s Office of Research Integrity and Assurance. Following IRB approval, participant recruitment commenced. Respondents were recruited via an online mommy blogger network, The Motherhood. To invite the sample of users to be part of this study, messages promoting the survey and inviting members of the network of mommy bloggers to participate were sent via the network manager via email. Three messages were posted including the original announcement and 2 follow-up messages to remind members to take the survey.

### Sample

The study analyzed survey responses by members of the online mommy blogger network, The Motherhood. The Motherhood is a leading online influencer network of more than 3000 members comprising moms as well as numerous other demographic groups that support campaign message dissemination for various initiatives such as health, consumer products, and entertainment [39]. The network taps into the need for human connection and focuses on building authentic relationships that benefit all involved [39]. The demographics of the network shift constantly but the following describe the composition of the network as provided by The Motherhood at the time of this study. Geographically, most members of the network are from the United States, with 1.18% in Canada. Across the United States, membership is fairly evenly distributed, with 20.9% in the Southeast; 7.42% in the Southwest; 12.58% in the West; 27.5% in the Northeast and East; 21.72% in the Midwest; and 8.7% in the Plains (personal communication by Erin Olson, August 23, 2016). The network is primarily female, with about 97% being women (personal communication by Erin Olson, August 23, 2016). This reflects the greater number of mom blogs in existence than dad blogs generally (personal communication by Erin Olson, August 23, 2016). The network also asserts that approximately 3% of the network is comprised of men (personal communication by Erin Olson, August 23, 2016). Again, ethnic breakdown continuously changes but recent data show 80% of the network is white, 8% Hispanic/Latino, 2.5% African American, 6% Asian, and 3.5% Other (personal communication by Erin Olson, August 23, 2016). Anyone with a blog and a following of at least 50,000 readers can request to join the network [39]. For this study, four hundred and sixty-one participants of the network initiated the online survey. 163 were removed for low quality responses and incomplete data. As a result, 298 eligible participants completed the survey.

### Measures

To collect data and evaluate the outcomes of this study, a 14-item survey was developed. All recruitment materials and messages included the link to the Web-based survey via Qualtrics. The first page of the survey contained informed consent materials. Participants clicked agree to provide consent. Participants answered sociodemographic questions as well as questions about key variables: Likelihood to share information; Topics of Conversations; and Motivations for sharing.

#### Likelihood to Share Information

Blogger likelihood to share health information was assessed by asking the question, “Would you consider writing about health topics in the future?” and a dichotomous two-point (Yes or No) scale.

#### Topics of Conversations

Blogger health topic preferences was assessed by asking the questions, “Which health issues did you write about on your blog?” and “Which health issues would you consider writing about on your blog?,” and providing a list of answer choices that included items such as Nutrition, Cancer, Vaccination, and Mental Health. These were selected from prior research [36]. Respondents could choose multiple responses.

#### Motivations for Sharing

To assess blogger motivations for sharing health information on their blog, the open-ended question, “What would motivate you to consider writing about health issues on your blog?,” was asked and respondents could provide a response in their own words. Responses were coded, analyzed, and then grouped into categories based on similarity of response.

The survey can be found in Multimedia Appendix 1.

### Analytical Process

The survey was developed and fielded using Qualtrics software. It was conducted from October 1 to October 28, 2016. Frequencies and chi-square tests were run using SPSS Statistical Software Version 20 to answer research questions 1, 2, and 6. A number of questions had open-ended responses, which is why themes were identified through both inductive and deductive processes [40,41], with themes and patterns from prior research with this community used [37,42] and additional themes that emerged from the interview data captured and added to the code frame throughout the data analysis process. Open-coding of the content was conducted on a random sample of 20% of the total number of responses [43]. Content coding was used to answer questions 3, 4, and 5. Two independent coders used this code frame to code responses to the open-ended questions; results were compared and interrater reliability was 90%.

## Results

The final sample size recruited for this study was 298 respondents. Among the respondent sample (N=298), the average age was 38 years. While 84.2% of the respondents (251/298) were under the age of 45 years, there were no respondents over the age of 65. In addition, 99.0% of the respondents reported being female (295/298), with males representing only 1% (3/298) of the respondent sample. Similarly, 99.0% of the respondents (295/298) reported having children while 1% (3/298) reported not having children. Additionally, 80.9% of the respondents (241/298) reported that they have histories of health issues while 19.1% (57/298) reported that they do not.

In terms of race, 81.2% (242/298) respondents were white. African Americans represented 5% (16/298) of the respondents; Hispanic/Latinos represented 9% (26/298) of the respondents; Asians represented 1% (4/298) of the respondents; and 3% (10/298) of the respondents chose to identify themselves as Other. Because of the low representation of non-white respondents, all other ethnicities were grouped into an Other category and whites and non-whites were compared for this analysis.

In terms of education, 50.3% of the respondents were college graduates (150/298) and 20% reported having a graduate degree (60/298). These 2 groups comprised 70.5% (210/298) of the sample. The remainder of the respondent sample was comprised as follows: 23% reported having some college education (68/298), 6% reported having a high school degree or GED (18/298), and 1% reported having some high school or less education (2/298). Because of the low representation from less well-educated bloggers, College Degree and Graduate Degree were combined and Some College, High School Degree, and High School or Less were combined. Table 1 includes frequency distributions for these variables.

The primary research question for this study explored how willing mommy bloggers are to discuss health issues on their blogs. In order to assess this question, basic frequencies tests were run on 2 questions in the survey—first, whether respondents have written about health information on their blogs in the past and whether they would consider reporting about health again in the future. Overall, 87.2% of the respondents (260/298) reported having written about health issues in the past. As well, 97.3% of the respondents (290/298) reported that they would consider writing about health issues sometime in the future. This is despite approximately 13% of the respondents (38/298) reporting that they had not written about health issues in the past. Table 2 details these frequencies.

The study’s second research question explored bloggers’ perceptions of their readers’ willingness to read about health issues on their blogs. In order to assess this question, a frequencies test was run on the question in the survey that asked the bloggers to assess their readers’ interest in reading about health information on their blogs. The majority of the respondents (96.3%) reported that their readers like to read about health issues on their blogs (287/298). Table 2 details these frequencies.

The third research question explored the topics bloggers had written about and what topics would be of interest to them in the future. In order to assess this question, a sample of responses (chosen from a list of topics selected from prior research [36]) to the question about which topics bloggers have written about were coded and analyzed. Respondents could choose multiple answers. Less than one-fourth (23.9%, 38/159) of the respondents reported having written about the topic of Physical Activity. In addition, 21.4% (34/159) of the respondents reported having written about the topic of Nutrition. Finally, 13.8% (22/159) of the respondents reported having written about the topic of Mental Health. Table 3 provides details on the topics.

In terms of what topics would be of interest to bloggers in the future, a sample of responses (chosen from a list of topics selected from prior research [36]) to the question about which topics bloggers would like to write about in the future were coded and analyzed. Respondents could choose multiple answers. Similarly, Physical Activity and Nutrition remain top content priorities for these bloggers for the future with 18.9% (54/286) reporting wanting to write about Physical Activity in the future and 17.8% (51/286) reporting wanting to write about Nutrition in the future. Mental Health is also a future interest for these bloggers with 14.3% (41/286) reporting wanting to write about this topic in the future. Finally, Heart Disease is also a topic of interest for these bloggers in the future, with 12.2% (35/286) reporting wanting to write about Heart Disease in the future. Table 3 provides details on the topics.

The fourth research question this study explored blogger perceptions about was which topics their readers would be interested in. In order to assess this question, a sample of responses (chosen from a list of topics selected from prior research [36]) to the question about which topics bloggers perceive their readers to be interested in were coded and analyzed. Respondents could choose multiple answers. Fifty-one respondents (21.3%, 51/239) reported that their readers would be interested in the topic of Nutrition; 21.3% (51/239) of the respondents reported that their readers would be interested in the topic of Physical Activity; and 16.7% (40/239) of the respondents reported that their readers would be interested in the topic of Mental Health. Finally, 10.9% (26/239) of the respondents reported that their readers would be interested in the topic of Vaccination. Table 3 details these data.

**Figure 1:**
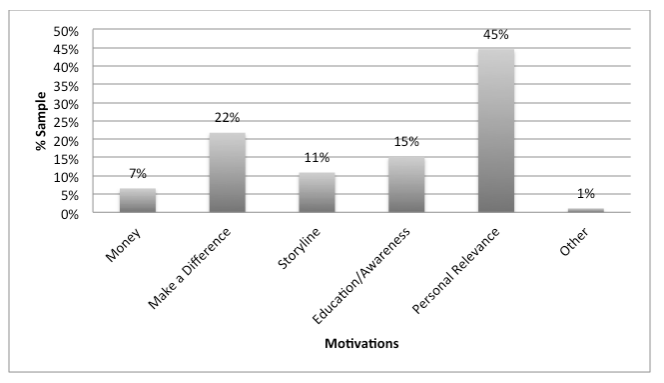
Motivations for blogger sample to write about a health issue.

The fifth research question this study aimed to explore was about what motivates bloggers to write about health issues. To assess this question, a sample of responses to the open-ended question about what motivates bloggers to write about health topics was coded and analyzed. The main motivation bloggers reported for supporting dissemination of health information is having a personal connection to the issue. Forty-one respondents (45%, 41/92) said that they would write about an issue if they had a connection to it, and 20 (22%, 20/92) reported that making a difference was a motivating factor. Finally, 15% (14/92) of the respondents reported that the desire to help educate or make people aware of an issue was a motivating factor. Only 7% (6/92) reported that compensation was a motivating factor. Figure 1 depicts these data.

The sixth, and final, research question this study aimed to explore focused on what factors contribute to bloggers writing about health issues. Using a chi-square test, the relationship between history of health issues and whether a blogger has written about a health issue in the past was assessed and found to be statistically significant, with χ^2^_1_ (n=298)=18.47, *P*<.001. This indicates that having a personal connection with a health issue is positively associated with likeliness to write about health issues on their blog.

**Table 1:** Frequency distributions for key variables in sample (N=298).

Variable		n (%)
**Age in years^a^**		
	18-44	251 (84.2)
	45+	47 (15.8)
**Gender**		
	Male	3 (1.0)
	Female	295 (99.0)
**Have children**		
	Yes	295 (99.0)
	No	3 (1.0)
**Have history of health issues**		
	Yes	241 (80.9)
	No	57 (19.1)
**Race/Ethnicity**		
	White	242 (81.2)
	Nonwhite	56 (18.8)
**Education**		
	Less than college	88 (29.5)
	College/graduate degree	210 (70.5)

^a^Individual ages were grouped in this chart based on US Census age groups (US Census, 2010)

**Table 2:** Health issue perspectives (N=298).

Variable		n (%)
**Written about health issues**		
	Yes	260 (87.2)
	No	38 (12.8)
**Willingness to write about health issues**		
	Yes	290 (97.3)
	No	8 (2.7)
**Blog reader interest in health issues**		
	Yes	287 (96.3)
	No	11 (3.7)

**Table 3:** Blog topic analysis in sample.

Variable		n (%)
**Topics written about**		
	Cancer	19 (11.9)
	Nutrition	34 (21.4)
	Physical Activity	38 (23.9)
	Diabetes	10 (6.3)
	Heart Disease	10 (6.3)
	Mental Health	22 (13.8)
	Vaccination	10 (6.3)
	Other	16 (10.1)
**Possible future topics**		
	Cancer	34 (11.9)
	Nutrition	51 (17.8)
	Physical Activity	54 (18.9)
	Diabetes	34 (11.9)
	Heart Disease	35 (12.2)
	Mental Health	41 (14.3)
	Vaccination	27 (9.4)
	Other	10 (3.5)
**Topics blog readers are interested in**		
	Cancer	24 (10.0)
	Nutrition	51 (21.3)
	Physical Activity	51 (21.3)
	Diabetes	19 (7.9)
	Heart Disease	22 (9.2)
	Mental Health	40 (16.7)
	Vaccination	26 (10.9)
	Other	6 (2.5)

## Discussion

### Overall Findings

This formative study attempted to capture the complexity of mommy bloggers’ perceptions about writing about health-related topics on their blogs, specifically, mommy blogger perspectives about which health issues are more likely to be written about and their motivations for doing so, as well as the perceived interest among their readers for consuming health-related content. In doing so, this study presents data that can inform future public health research and communication campaigns to more broadly promote their messages, thereby contributing to their behavior change objectives. The findings from the analysis of the sample for this study are worthy of discussion and hold several implications for future work in this area. The following in-depth discussion of the study’s results begins an exploration of how this work contributes theoretical and translational insights to the social science literature.

In terms of the sample, in this study, the sample was relatively homogenous. It was both a younger group as well as a well-educated one. It skewed white, with less than 20% of the respondents being non-white. Most of these bloggers have children and have dealt with health issues themselves. This study’s findings showed that 87% of the respondents reported that they have written about health issues in the past and that 97% of the respondents reported that they would consider writing about health issues sometime in the future. Additionally, 96% of the respondents reported that their readers like to read about health issues on their blogs with the topics of Nutrition and Physical Activity being most commonly cited as having been written about by the respondent sample. Moreover, a significant interaction was found between having a history of health issues and likeliness to write about health issues (*P*<.001). Having a family history of health issues was found to be positively associated with likeliness to write about health issues on a blog (*P*<.001).

### Theoretical Implications

Theoretically, this study contributes to the literature in a number of ways. First, as noted earlier, there is limited work in the area of mommy bloggers as communication channels for health information to-date. As Lee et al [44] contend, “This popularity has not been fully reflected in empirical academic research yet. Broader publications have considered the influence of blog(ger)s on politics and mass communications [45], tourism [46], journalism [47], and public relations [48].”

In addition, Sundar et al [25] suggest, “Relatively little media and scholarly attention has been paid to personal blogs that discuss topics other than politics. For example, the burgeoning number of health blogs has gone virtually unnoticed in mainstream media and the academic discourse about blogs.”

Specific prior research in this area has included work conducted by Burke-Garcia et al where mommy bloggers participated in formative research, followed by an intervention, in order to understand motivations and barriers as well as reader reactions to sharing vaccination information on their blogs [36]. Findings revealed key barriers that bloggers face in sharing vaccination information but that despite these barriers, bloggers who do share this information can drive health behavior consideration among their readers [36]. As well, Horn et al’s pilot study on use of mommy bloggers to reach communities of color with health information found that the collaboration between health professionals and social media leaders in communities of color has the ability to influence content and dialogue and thus may be able to help reduce health disparities [37].

This study’s findings expand on this current knowledge base by conducting research with a larger sample of this population, looking at perceptions of writing about health topics more broadly (not specifically vaccination), and providing updated data on Web-based health information consumption and dissemination patterns by mommy bloggers to their readers. The findings that mommy bloggers are willing to share health information and their perceptions of their readers’ interests in consuming this kind of information via blogs are substantial because as text-based, photo, or video content is being created and shared at an unprecedented level on these blogs—which includes individuals’ opinions and ideas—this community has grown to have a virtual finger on the pulse of the needs and beliefs of their Web-based audiences [23]. Therefore, they can provide a window into the needs and wants for their readers and act as trusted communication channels for evidence-based health information on a wide variety of health topics to be communicated to them in ways that work for them. This is worthy of more exploration and comprehension as public health professionals and other scholars seek to understand how to engage with this audience to achieve their program goals.

Finally, while understanding the point of view of bloggers may be theoretically interesting, it also may have implications for real life. Blogs are imbued “with a strong sense of the author’s personality, passions, and point of view” [24], and provide the writers’ own points of view [49,50]. This may lead them to write about health issues in ways that are problematic for their readers. Risk and Petersen [51] posit, “A plethora of inaccurate and even potentially life-threatening content readily accessible to anyone with a modem and an Internet browser supports the validity of that concern. For instance, Crocco, Villasis-Keever and Jadad reported that inaccurate Internet information contributed to harm in a 1-year-old boy with diarrhea.”

### Translational Implications

Thus these results also hold promise for future translational health communication efforts. First, there is the potential to work in collaboration with bloggers to promote dissemination of relevant health information. Given the data that suggest the influence these bloggers have with other moms, parents, and caregivers, these should be considered as part of any future health communication campaign seeking to reach and engage these audiences. Additionally, since the data suggest that a primary motivation for bloggers to write about health issues is having a personal connection with the health issues, it indicates that it will be important for public health communicators to get to know bloggers so they can pitch blogging interventions concerning health issues that are most salient to specific bloggers. It also means that the health topic is a critical consideration for bloggers when deciding whether or not to support a campaign. The storyline for the health issue will also impact blogger engagement. Ultimately, the topic of the issue and how it is communicated to bloggers is paramount to bloggers’ decisions about whether or not to write about the issue.

This study illustrates that there are potentially rich opportunities for working with mommy bloggers to communicate with key health decision makers (moms) about important health issues. Mommy bloggers are interested in writing about these important health issues and their audiences of moms are interested in reading about these issues. Moreover, the blogs can provide new interactive Web-based communication channels for bloggers and their audiences to communicate about health issues, answer questions, and provide needed support for making health decisions. By working with bloggers to disseminate health information, health communicators can leverage the established powerful, well-utilized, and trusting online social networks established on mommy blogs, while also providing bloggers with relevant, accurate, and up-to-date health information to share with their audiences. This Web-based communication strategy can build upon the established relational strength and influence of communication between bloggers, while also helping to ensure that the health information provided via the blogs is of high accuracy and quality. Ultimately, the value of these mommy blogs should not be overlooked by public health communicators and should be further explored as sources of interpersonal influence concerning important health issues.

### Limitations and Future Research

A constraint of this study was the time frame for study design, data collection, and analysis. In addition, there was not an opportunity to gain additional insight by surveying readers of blogs and through interviews with the bloggers themselves. Additionally, this study made use of cross-sectional, self-reported data, among an opt-in sample with the possibility of self-selection bias. This may have skewed how respondents responded to the questions and therefore the data gathered. Finally, the sample skewed white, educated, female, and younger, which may have influenced the results.

Future research should aim to explore this topic with a more diverse sample including men, more representatives from different race or ethnicities, less well-educated bloggers, as well as readers of blogs themselves. There is clearly a need to identify and work with a more diverse sample of mommy bloggers to more broadly and thoroughly disseminate tailored and targeted health information via these trusted networks. Finally, future research should build in interviews or focus groups to gather insights directly from the bloggers in order to better understand their attitudes and motivators and how communication initiatives can work with them in the future.

## Appendix

Multimedia Appendix 1 Survey Instrument.

## Abbreviations

IRB: institutional review board
