# Supplementary material for: Perceptions About Disseminating Health Information Among Mommy Bloggers: Quantitative Study
Source: JMIR Res Protoc. 2018 Apr 24;7(4):e116. doi: 10.2196/resprot.7764 (PMC5941100; doi:10.2196/resprot.7764)
Supplement: Multimedia Appendix 1 [file resprot_v7i4e116_app1.pdf]

Instrument

1. Have you written about health issues on your blog?

- Yes/no

1a. If yes, which health issues did you write about on your blog? (Choose all that apply)

- Cancer
- Nutrition
- Physical activity
- Diabetes
- Heart disease
- Mental health
- Vaccination
- All of the above
- Other [fill in]

2. Would you like to write about relevant health topics more often on your blog?

- Yes/no

3. Which health issues would you consider writing about on your blog? (Choose all that apply)

- Cancer
- Nutrition
- Physical activity
- Diabetes
- Heart disease
- Mental health
- Vaccination
- All of the above
- Other [fill in]

4. What would motivate you to consider writing about health issues on your blog?

[open]

5. Do you think your readers like to read posts about health issues on your blog?

- Yes/no

6. Which health topics do you think your blog readers would be interested in? (Choose all that apply)

- Cancer
- Nutrition
- Physical activity
- Diabetes
- Heart disease
- Mental health
- Vaccination
- All of the above
- Other [fill in]

Please share some of your background information:

7. What is your age?

[open, but limited to 2 characters]

8. What is your gender?
- Male
- Female
- Other

9. Do you have children?

- Yes/no

10. Do you have a family history of health issues?

- Yes/no

10a. If yes, which health issues have you or your family experienced? (Choose all that apply)

- Cancer
- Obesity
- Diabetes
- Heart disease
- Mental health
- All of the above
- Other [fill in]

10b. Please describe your experience with any of these health issues.

[open]

11. What is the zip code of where you live?

[open]

12. How would you describe yourself?

- White/Caucasian
- African American
- Hispanic/Latino
- Asian
- Other

13. What is the highest level of education you have obtained?

- High school or less
- High school degree or GED
- Some college
- College degree
- Graduate degree

14. Do you have any additional comments?

[open]
